# Association of personalised care plans with monitoring and control of clinical outcomes, prescription of medication and utilisation of primary care services in patients with type 2 diabetes: an observational real-world study

**DOI:** 10.1080/02813432.2022.2036458

**Published:** 2022-02-11

**Authors:** Ilona Mikkola, Simon Morgan, Klas Winell, Jari Jokelainen, Lucia Frittitta, Eveliina Heikkala, Maria Hagnäs

**Affiliations:** a Rovaniemi Health Centre, Rovaniemi, Finland; b Elermore Vael General Practice, Elermore Vale, Australia; c Conmedic, Espoo, Finland; d Infrastructure for Population Studies, Faculty of Medicine, University of Oulu, Oulu, Finland; e Unit of General Practice, Oulu University Hospital, Oulu, Finland; f Endocrinology, Department of Clinical and Experimental Medicine, University of Catania, Catania, Italy; g Diabetes, Obesity and Dietetic Center, Garibaldi-Nesima Medical Center, Catania, Italy; h Center for Life Course Health Research, University of Oulu, Oulu, Finland

**Keywords:** Goal achievement, patient-centric care, general practice services, care plan, type 2 diabetes

## Abstract

**Objective:**

To study the association of personalised care plans with monitoring and controlling clinical outcomes, prescription of cardiovascular and antihyperglycaemic medication and utilisation of primary care services in patients with type 2 diabetes (T2D).

**Patients:**

Primary care T2D outpatients from the Rovaniemi Health Centre.

**Setting:**

The municipal health centre, Rovaniemi, Finland.

**Design:**

A cross-sectional, observational, retrospective register-based study. The patients were divided into three groups: ‘no care plan entries’ (usual care); ‘1–2 care plan entries’; and ‘3 or more care plan entries’.

**Main outcome measures:**

Monitoring of clinical and biochemical measures, achievement of treatment targets, prescription of cardiovascular and antihyperglycemic medication, and use of primary care services.

**Results:**

A total of 5104 patients with T2D (mean age 65.5 years (SD 12.4)), of which 67% had at least one care plan entry. Compared to usual care, the establishment of a care plan (either care plan group) was associated with better monitoring of glycosylated haemoglobin A1c, low-density-lipoprotein cholesterol, systolic blood pressure (sBP), and renal function, and there was more frequent prescription of all cardiovascular and antihyperglycemic medication. Patients in either care plan group were more likely to achieve sBP target (*p* < 0.05). Patients without a care plan had more unplanned primary care physician contacts compared to patients in care plan groups (*p* < 0.001).

**Conclusion:**

Establishment of a care plan is associated with more intensive and focussed care of patients with T2D. The appropriate use of primary care resources is essential and personalised care plans may contribute to the treatment of patients with T2D.

**Key Points**
- Care planning aims to empower patients with type 2 diabetes. This study demonstrates that personalised care planning is associated with
- more frequent monitoring for clinical outcomes,
- more frequent prescription of cardiovascular and antihyperglycemic medication and
- more frequent utilisation of planned diabetes consultations when compared to usual care.

## Introduction

Type 2 diabetes (T2D), with its micro- and macrovascular complications, is an important cause of mortality and morbidity, and is a major economic burden worldwide [1]. Treatment of T2D is multidimensional and it requires control of glycaemia and cardiovascular risk factors, regular monitoring and follow-up, and a patient-centred approach. The usual provider of diagnosis and care for T2D in northern Europe is the primary care physician [2,3].

A personalised care plan is a formally documented, patient-centric, forward-looking, comprehensive series of discussions (one or several) between patient and care provider in which they collaboratively agree on goals and actions to manage the patient's condition. A care plan has been recommended in the management of T2D [4,5]. Compared to usual care, personalised care plans have been shown to lead to improvements in a variety of indicators of physical and psychological health, as well as a patient's capability to self‐manage their condition, including diabetes [6]. Care plans involve a range of potential interventions. Interventions integrated into routine care and those of higher intensity (i.e. more frequent follow-up) have been found to be particularly beneficial [6]. Generally, self-management interventions have not been found to be easy to adopt and implement in primary care [7].

In 2015, a personalised care plan for patients with T2D was successfully implemented in the Rovaniemi Health Center, Finland, using a breakthrough method (a collaborative improvement model) [8] and networking with the Finnish Quality Network [9]. Since then, it has been shown that implementation of a care plan is associated with small improvements in some clinical outcomes of T2D patients [10]. Additionally, other previous studies have shown that care planning is associated with higher prescription rates of metformin [11] and statins [9] among T2D patients. More studies on care planning in real-life settings have been called for [7]. To our knowledge, the association of care planning on the use of other cardiovascular medication and clinicians’ services has not been studied.

Therefore, we conducted a study investigating care planning in the real world. Our aim was to investigate the association between clinical and biochemical monitoring and outcome measures (blood pressure (BP), glycosylated haemoglobin (HbA1c), lipid levels, renal function and body mass index (BMI)) in patients with T2D, with the use of a personalised care plan. We additionally studied the prescription rate of cardiovascular and antihyperglycaemic medication, and the use of primary care physician services. We hypothesised that improved clinical and biochemical outcome measures and frequency of monitoring are associated with the number of care planning discussions. Furthermore, we hypothesised that care plan use is associated with more intensive medical treatment for T2D and a higher number of planned diabetes consultations but a decreased use of other ’ad hoc’ primary care services.

## Material and methods

### Patients

This study was part of the Rovaniemi Primary Care T2D Study, conducted in the municipal Rovaniemi Health Center, Rovaniemi, Finland. Rovaniemi has a total population of approximately 62 000 people residing in both urban and rural areas. There are 57 physician posts at the municipal Rovaniemi Health Center, including three primary care clinics.

Study subjects consisted of primary care T2D outpatients, whose data at baseline were available. We excluded patients who did not have at least one entry of any kind in their patient records during the follow-up period.

A diagnosis of T2D was defined as the presence in the record of codes E11.1–E11.9 for T2D according to tenth revision of the World Health Organization’s International Classification of Disease (ICD-10) [12] or code T90 according to the International Classification of Primary Care (ICPC) [13]. In Finland, diagnosis and routine care of patients with T2D are usually provided by primary care physicians, who work closely with the primary care nurses.

### Study protocol and data collection

The present study adopted a retrospective, real-world protocol with a cross-sectional and longitudinal design. Data were collected from patient records based on the prerequisite of a diagnosis of T2D. Study data consisted of information recorded as part of patients’ visits or treatment. Subjects were identified and administered only by their assigned study ID. Data were collected anonymously for scientific purposes only. The study protocol was approved by the Ethics Committee of the Lapland Central Hospital, Rovaniemi, Finland.

### Descriptions of baseline and follow-up period, duration of treatment and multimorbidity

The beginning of the treatment (the baseline) was defined for each patient as the date of the patient’s first consultation (whether the patient had been newly or previously diagnosed) during the period January 2011 to July 2017. The follow-up period was from August 2017 to February 2019. Duration of treatment was defined as the time period from baseline to the end of the follow-up period. In Rovaniemi Health Centre, the maximum time interval between routine visits is set at 1.5 years. Therefore, the cut-off point for the follow-up visits was set as July–August 2017, which is 1.5 years from the end of the data collection in February 2019.

Gender, age and the most common long-term conditions were collected from the patient records at baseline. ‘Multimorbidity’ was defined as a patient having at least one other long-term diagnosis (excluding hypertension and dyslipidaemia which were defined as ‘comorbidities’ of T2D).

### Care plan and usual care

The structured personalised care plan form is a tool designed for national use by the Finnish Institute for Health and Welfare. It aims to improve the care of long-term patients and to empower them to take care of their own health. The form can be implemented with a standard structure in all patient information systems and is thus also available regardless of the patient record system used [14]. Usually, establishment of the care plan includes different number of actions, which are called a care plan cycle. The care plan in Rovaniemi health centre includes all the recommended seven steps of the care planning cycle - preparation, goal setting, action planning, documenting, coordinating, supporting and reviewing [6]. A template of the care plan form is presented in detail in Supplement 1.

The care plan was integrated into the routine care of a small number of patients with T2D in Rovaniemi Health Centre in 2013, broader implementation occurred in 2015. Successful implementation was enabled by establishing the care plan template into the patient record, as well as routine utilisation of self-management forms. A minimum three-hour education programme for staff on the process was organised. While the care plan has a formal set of instructions around its use, our study protocol did not include standardisation of the implementation across all caregivers, reflecting the real-world setting.

As part of routine care, a self-management questionnaire is mailed to patients approximately two-four weeks prior to each planned diabetes consultation. The questionnaire included open-ended free-text questions about the patient's expectations for the visit and the desired goals of the treatment.

Additionally, structured forms for BP and glucose home monitoring were posted with the self-management questionnaires for completion by the patients. Patients were also instructed to list all current medication used. During the consultation, this information was collated and discussed with the patient to yield a shared decision about actions and medications, follow-up plan etc. needed.

All physicians and nurses were encouraged to offer a care plan to their patients as part of routine care, but not all individuals (either caregivers or patients) accepted. For the purposes of the study, those with no care plan entries were defined as the ‘no care plan entries’ group. Those with any care plan entries were divided into two groups: patients with one or two entries and patients with three or more (3+) entries. A care plan entry in Rovaniemi comprised pre-consultation preparations (e.g. laboratory tests and a self-care form), consultation and establishment of the care plan

### Outcome measures

The following clinical variables were retrieved from patient records both at baseline and follow-up: systolic blood pressure (sBP), diastolic blood pressure (dBP) and body mass index (BMI). The preferred source of BP data was the patient’s own home measurements, from which the mean was calculated (BP is measured at home for four days, including morning and evening double measurements) [15]. If home measurements were not available, the measurements performed during the consultation visit were used.

The following biochemical variables were retrieved from patient records both at baseline and follow-up: plasma creatinine (µmol/l), eGFR (ml/min/1.73m^2^), HbA1c (mmol/mol), total serum cholesterol (mmol/l), high-density lipoprotein (HDL) cholesterol (mmol/l), low-density lipoprotein (LDL) cholesterol (mmol/l), triglycerides (mmol/l), urinary albumin-creatinine ratio (u-ACR) (mg/mmol), and overnight urine albumin excretion (cU-Alb) (µg/min). The dual measurement of renal function was defined as both eGFR and u-ACR or cU-Alb. The study protocol specified the following clinical target levels: home sBP < 135 mmHg, HbA1c < 53 mmol/mol, and LDL < 2.5 mmol/l, according to Finnish current care guidelines for T2D [16].

Prescription data for ongoing medications, based on Anatomical Therapeutic Chemical (ATC) codes were collected from patient records, both at baseline and follow-up. The number of contacts of each patient to primary care and other services was collected at follow up. Contacts were categorised as either ‘planned diabetes consultations’ or ‘other primary care physician contacts’. ‘Planned diabetes consultation’ described the prepared, double-timed physician or nurse consultation visits, which were allocated for patients needing long-term care. ‘Other primary care physician contacts’ included ‘physician consultations’ which were allocated for patients needing treatment for a subacute health problem, excluding emergency room visits. ‘Other’ means a physician service without a visit, i.e. a nurse consulting a physician, or a letter or telephone call to the patient.

### Statistical methods

Clinical outcome measures were presented as mean and standard deviation (SD), categorical variables were presented as proportions. Analysis of variance (ANOVA) was used to assess the significance of differences in continuous variables. A χ^2^-test was used to evaluate the difference between categorical values. The association of care plan entries (0, 1–2, 3+) with monitoring of clinical outcomes and presence of albuminuria, achievement of treatment targets, and prescription of cardiovascular and antihyperglycemic medication was examined using univariable and multivariable logistic regression. The regression model was built in two stages to observe changes in risk estimates. The adjusted model included length of treatment period (at the end of the follow-up), age (at the time of diagnosis), sex and baseline values for current variable. The results are reported as odds ratios (ORs) with 95% confidence intervals (95% CIs). Both unadjusted and adjusted estimates are presented.

Statistical analyses were performed using IBM SPSS Statistics for Macintosh, Version 24.0. Armonk, NY: IBM Corp. IBM Corp. Released 2016. A *p*-value < 0.05 was considered statistically significant.

## Results

### Baseline

A total of 5104 patients were included in the analyses, 54% of whom were male. The mean age of the study population was 65.5 years (SD 12.4) at baseline. The mean BMI at baseline was 30.3 kg/m^2^ (SD 6.0) (Table I). In addition to being diagnosed with T2D, one patient was wrongly diagnosed also with ‘gestational diabetes’, and 108 with ‘type 1 diabetes’. Of all patients, 93.3% were defined as multimorbid. The proportion of patients with depression or cancer was 4.2% and 9.35%, respectively, and there was no statistical difference in their prevalence between the groups. (data not shown)

**Table 1.** Baseline characteristics of the study population by care plan groups (categorized at follow-up as 0, 1–2, and 3 or more care plan entries).

			Care plan entries					
	Total	Monitored	0	Monitored	1–2	Monitored	≥ 3	Monitored
	5104 (100%)		1709 (33.5%)		1734 (34.0%)		1661 (33.0%)	
Gender								
Male	2755 (54.0%)		866 (50.7%)		966 (55.7%)		923 (55.6%)	
Female	2349 (46.0%)		843 (49.3%)		768 (44.3%)		738 (44.4%)	
Age	65.5 (12.4)		67.9 (14.4)		64.3 (11.7)		64.3 (10.3)	
Weight, kg	84.5 (19.2)	62.2%	80.6 (20.6)	59.2%	86.2 (18.6)	58.6%	86.3 (17.9)	69.0%
BMI, kg/m2	30.3 (6.0)	61.4%	29.6 (6.5)	55.2%	30.4 (5.8)	58.0%	30.6 (5.7)	71.2%
sBP, mmHg	142 (21.6)	62.2%	139 (22.6)	66.6%	145 (22.0)	57.0%	143 (19.7)	63.1%
dBP, mmHg	80.3 (12.1)	62.1%	78.3 (13.2)	66.6%	82.0 (11.9)	56.8%	80.8 (10.9)	63.0%
B-Hb, g/l	141 (14.7)		137 (16.5)		144 (13.4)		143 (13.2)	
HbA1c, mmol/mol	51.4 (14.6)	89.3%	52.7 (15.9)	83.4%	48.6 (12.1)	86.5%	53.0 (15.1)	98.1%
Total serum cholesterol, mmol/l	4.8 (1.2)	71.4%	4.8 (1.3)	62.1%	4.9 (1.2)	72.8%	4.8 (1.2)	79.4%
HDL cholesterol, mmol/l	1.3 (0.4)	62.3%	1.3 (0.4)	51.7%	1.3 (0.4)	62.5%	1.3 (0.3)	73.1%
LDL cholesterol, mmol/l	2.8 (1.0)	87.5%	2.8 (1.1)	79.5%	2.9 (1.1)	85.3%	2.8 (1.0)	98.1%
Triglycerides, mmol/l	1.8 (1.3)	59.7%	1.8 (1.5)	48.9%	1.8 (1.2)	60.0%	1.8 (1.3)	70.6%
eGFR, ml/min/1.73m2	77.7 (19.7)	77.3%	72.5 (23.2)	57.5%	79.4 (18.5)	79.3%	79.5 (17.7)	95.7%
P-creatinine, µmol/l	80.9 (32.0)	91.9%	86.5 (48.1)	88.4%	78.7 (19.8)	89.1%	77.8 (19.6)	98.5%
U-ACR, mg/mmol	5.14 (26.7)	50.6%	10.4 (44.6)	50.6%	3.38 (17.5)	62.1%	3.23 (14.8)	84.2%
cU-alb, µg/min	29.4 (140)	73.9%	50.8 (175)	56.1%	19.3 (70.8)	72.5%	24.3 (156)	93.7%
Cardiovascular preventive medication								
ACE inhibitors	1557 (30.5%)		483 (28.3%)		505 (29.1%)		569 (34.3%)	
ARB	1367 (26.8%)		391 (22.9%)		451 (26.0%)		525 (31.6%)	
Beta blockers	2269 (44.5%)		788 (46.1%)		710 (40.9%)		771 (46.4%)	
Calcium blockers	1840 (36.1%)		526 (30.8%)		590 (34.0%)		724 (43.6%)	
Diuretics	1641 (32.2%)		720 (42.1%)		435 (25.1%)		486 (29.3%)	
Statins	3357 (65.8%)		903 (52.8%)		1146 (66.1%)		1308 (78.7%)	
Ezetimibe	347 (6.80%)		70 (4.10%)		122 (7.04%)		155 (9.33%)	
Fibrates	28 (0.55%)		4 (0.23%)		12 (0.69%)		12 (0.72%)	
Antihyperglycaemic medication								
Metformin	3199 (62.7%)		809 (47.3%)		1101 (63.5%)		1289 (77.6%)	
SGLT-2 inhibitors	185 (3.6%)		17 (1.0%)		61 (3.5%)		107 (6.4%)	
DPP-4 inhibitors	1295 (25.4%)		451 (26.4%)		348 (20.1%)		496 (29.9%)	
GLP-1 analogues	37 (0.7%)		5 (0.3%)		9 (0.5%)		23 (1.4%)	
Insulins	1101 (21.6%)		412 (24.1%)		212 (12.2%)		477 (28.7%)	
Glitazones	18 (0.4%)		2 (0.1%)		5 (0.3%)		11 (0.7%)	
Sulphonylureas	196 (3.8%)		64 (3.7%)		61 (3.5%)		71 (4.3%)	

Data are presented mean (SD), except for medication (given as percentage of the population). ACE: angiotensin-converting enzyme; ARB: angiotensin receptor blockers; BMI: body mass index; cU-alb: overnight urine albumin secretion; dBP: diastolic blood pressure; DPP-4: dipeptidyl-peptidase-4; eGFR: estimated glomerular filtration rate; GLP-1: glucagon-like peptide-1; Hb: hemoglobin; HbA1c: glycosylated haemoglobin; HDL: high-density lipoprotein; LDL: low-density lipoprotein; sBP: systolic blood pressure; SGLT-2: sodium-glucose co-transporter-2; T2D: type 2 diabetes; U-ACR: urinary albumin-creatinine ratio.

The baseline characteristics and use of medication of the patients by care plan groups are shown in Table 1. One-third of all patients had no care plan entries. The patients in the ‘no care plan’ group were significantly older, had lower BMI and BP and lower haemoglobin (Hb) and eGFR levels compared to any care plan entry groups at baseline (*p* < 0.05). On the other hand, the patients in group of 1-2 care plans had higher sBP, dBP, HbA1c, cholesterol and LDL levels compared to group of 3+ care plans (*p* < 0.05 for all). The proportion of patients who had already been prescribed cardiovascular and antihyperglycemics at baseline was lower in the ‘no care plan group’ than in the care plan groups for almost all medications (Table 1). However, despite being older and having antihypertensives infrequently prescribed, the patients in the no care plan group had lower BP at baseline compared to either care plan group.

### Follow-up

Monitoring of the clinical and biochemical parameters, achievements of treatment targets, and the change of use of cardiovascular and antihyperglycemic medication are presented in Table 2. The mean duration of follow-up was 5.1 years (SD 2.3). Monitoring of HbA1c and LDL levels, and dual tests of renal function was significantly more frequent in the care plan groups compared to the no care plan group (*p* < 0.05 for all). Monitoring of these parameters was significantly more frequent in group with 3+ care plan entries compared to patients with one or two entries (Table 2). After adjustment, there was a significant difference for sBP target achievement favouring care plan groups, and for LDL target achievement favouring care plan group 3+ entries. The rate of prescription for cardiovascular and antihyperglycemic medication was significantly higher in both care plan groups compared to the ‘no care plan’ group across the studied medicines. Antihypertensives, statins and antihyperglycemic medications were significantly more prescribed in care plan group 3+ entries than in group with one or two entries (Table 2).

**Table 2.** Association between establishment of personalised care plan (0, 1–2, 3 or more care plan entries) and monitoring of clinical outcomes and achievement of treatment goals and use of preventive cardiovascular and antihyperglycemic medication in primary care patients with type 2 diabetes at follow-up.

	Crude				Adjusted			
	1-2 care plan entries		≥3 care plan entries		1-2 care plan entries		≥ 3 care plan entries	
	OR (95%CI)	*p*	OR (95%CI)	*p*	OR (95%CI)	*p*	OR (95%CI)	*p*
Monitoring of clinical outcomes								
sBP monitored	1.06 (0.90 − 1.24)	0.490	0.96 (0.82 − 1.12)	0.620	1.21 (1.03 − 1.44)	0.030	1.02 (0.86 − 1.21)	0.850
HbA1C monitored	6.55 (5.44 − 7.90)	<0.001	21.12 (16.40 − 27.55)	<0.001	6.91 (5.72 − 8.37)	<0.001	23.58 (18.15 − 31.02)	<0.001
LDL monitored	6.66 (5.577.97)	<0.001	18.14 (14.50 − 22.87)	<0.001	6.68 (5.58 − 8.01)	<0.001	20.54 (16.28 − 26.13)	<0.001
Dual renal function monitored	6.32 (5.29 − 7.57)	<0.001	13.30 (11.02 − 16.11)	<0.001	6.25 (5.23 − 7.50)	<0.001	12.95 (10.68 − 15.75)	<0.001
Treatment goals								
sB*p* < 135 mmHg	1.29 (1.03 − 1.62)	0.030	1.35 (1.08 − 1.70)	0.010	1.32 (1.01 − 1.74)	0.050	1.34 (1.02 − 1.76)	0.040
HbA1*C* < 53 mmol/mol	1.69 (1.32 − 2.16)	<0.001	0.75 (0.59 − 0.94)	0.020	1.53 (1.11 − 2.10)	0.010	1.12 (0.83 − 1.50)	0.460
LD*L* < 2.5 mmol/l	0.90 (0.72 − 1.11)	0.330	1.52 (1.22 − 1.89)	<0.001	0.97 (0.74 − 1.25)	0.800	1.34 (1.03 − 1.74)	0.030
Albuminuria	1.51 (1.07 − 2.10)	0.020	1.13 (0.81 − 1.56)	0.460	1.45 (0.90 − 2.29)	0.130	1.26 (0.79 − 1.95)	0.320
Medication								
Antihypertensive medication of any kind	1.86 (1.56 − 2.22)	<0.001	4.06 (3.32 − 4.97)	<0.001	2.16 (1.79 − 2.61)	<0.001	4.27 (3.44 − 5.32)	<0.001
ACE inhibitors	1.24 (1.03 − 1.50)	0.030	1.50 (1.25 − 1.81)	<0.001	1.26 (1.04 − 1.52)	0.020	1.45 (1.20 − 1.75)	<0.001
ARB	1.49 (1.24 − 1.79)	<0.001	1.75 (1.46 − 2.10)	<0.001	1.60 (1.32 − 1.93)	<0.001	1.85 (1.53 − 2.24)	<0.001
Statins	2.44 (2.08 − 2.86)	<0.001	3.82 (3.24 − 4.51)	<0.001	2.61 (2.21 − 3.07)	<0.001	3.79 (3.19 − 4.51)	<0.001
Antihyperglycemic medication of any kind	3.33 (2.82 − 3.94)	<0.001	6.97 (5.79 − 8.42)	<0.001	3.37 (2.85 − 3.99)	<0.001	6.55 (5.41 − 7.95)	<0.001
Metformin	3.37 (2.86 − 3.97)	<0.001	4.28 (3.63 − 5.06)	<0.001	3.33 (2.83 − 3.93)	<0.001	4.67 (3.94 − 5.56)	<0.001
SGLT-2 inhibitors	2.47 (1.85 − 3.34)	<0.001	4.67 (3.54 − 6.25)	<0.001	2.51 (1.87 − 3.42)	<0.001	4.10 (3.08 − 5.55)	<0.001
Insulins	0.79 (0.63 − 0.99)	0.040	2.33 (1.91 − 2.86)	<0.001	0.79 (0.63 − 1.00)	0.050	1.64 (1.33 − 2.03)	<0.001

Comparison to usual care group (as 0 care plan entries).

ACE: angiotensin-converting enzyme; ARB: angiotensin receptor blockers; HbA1c: glycosylated hemoglobin; LDL: low-density lipoprotein; sBP: systolic blood pressure; SGLT-2: sodium-glucose co-transporter-2.

Table 3 presents the association between establishment of a care plan and primary care service utilisation in follow-up. Patients in the ‘no care plan’ group had significantly fewer ‘planned diabetes consultations’ but had more other primary care physician contacts compared to both care plan groups (*p* < 0.05 for both comparisons).

**Table 3.** Association between establishment of personalised care plan (0, 12, 3 or more care plan entries), and number of primary care contacts in patients with type 2 diabetes in follow-up.

		Care plan entries						
	Total	0	1–2	≥ 3	*p* Value			
	4309	1037 (24.1%)	1633 (37.9%)	1639 (38.0%)	Overall	0 vs. 1-2	1-2 vs. ≥ 3	0 vs. ≥ 3
Planned diabetes consultations	3.5 (1.6)	2.3 (1.2)	3.3 (1.5)	3.9 (1.7)	<0.001	<0.001	<0.001	<0.001
Other primary care physician contacts	7.7 (7.6)	10.6 (11.0)	7.1 (6.6)	6.7 (5.8)	<0.001	<0.001	0.085	<0.001
Physician consultations	1.4 (1.8)	1.3 (1.9)	1.4 (1.7)	1.4 (1.8)	0.556	0.315	0.910	0.366
Other contacts	5.4 (5.9)	7.7 (8.9)	4.9 (5.0)	4.5 (4.0)	<0.001	<0.001	0.031	<0.001

Data are presented mean (SD). *P* values present the differences between groups tested with ANOVA.

The care plan groups also differed from each other at baseline (Table 1). Patients with 3+ care plan entries had lower BP and LDL and higher HbA1c and they used more cardiovascular and antihyperglycemic medication compared to patients with one or two entries.

## Discussion

### Statement of principal findings

We found that the use of a care plan was associated with more frequent monitoring of HbA1c, LDL and BP levels, and dual renal function and more frequent prescription of cardiovascular and antihyperglycemic medication; thus, there was achievement of some of the treatment targets in patients with T2D in a real-world setting. The results improved with an increasing number of care plan entries. Additionally, we found that care plan use was associated with greater uptake of planned diabetes consultations, and fewer other primary care physician contacts compared with usual care.

### Strengths and weaknesses of the study

The most important strength of the present study is that it employs a large and representative number of primary care patients with T2D. The patient record data in Finland are reliable.

The real-world setting is both a strength, and a weakness of the present study. As a pragmatic real-world study, it does not require the need to translate evidence from controlled randomised trials into real-world primary care settings. The most important limitation of this study is that it is a retrospective, observational, real-world study setting; this leads to a lack of randomisation. Only associations- not causality- are possible to report.

We do not know why about one-third of the patients did not have a written care plan, even though they were certainly recommended to everyone. It may be that the health-care professional and the patient could not agree, or that the health professional judged that it would not benefit care [17]. Interpersonal factors, motivation, organisational factors and external factors have previously been proposed as explanations for lack of care plan [17,18]. Additionally, we can gain some understanding of the reason in our study by comparing the results in the entries of the three care plan groups. Research is needed to further study the characteristics of the patients in the no care plan group to understand the underlying causes (e.g. co-existing diseases and socio-economic status) of not having a care plan. Additionally, analysis of the situations and reasons why caregivers were not able to offer a recommended intervention to their patients should be recognised

The study was limited by being performed in a single centre. However, this can also be seen as a strength, as processes in the one health centre are similar. In a single-centre study, the beneficial care-plan-related practices may also have spread into the ‘no care plan’ group. This decreases the likelihood of the results being overestimated. Additionally, the context of real-life care plans and discussions have not been verified; thus, the beneficial aspect of the care planning process still remains unclear, which can also be considered a limitation. This study was also limited by its lack of data on whether the patients measured their BP regularly at home or had their BP measured only during the consultation. Moreover, all the caregivers do not routinely mark BP or BMI in structured way in the patient record system, which might also have affected our results concerning BP and BMI monitoring frequency. The number of confounding factors which may have influenced the clinical outcomes is unlimited (e.g. socio-economic factors, educational level, marital status and self-efficacy).

### Findings in relation to other studies

We found that the monitoring of clinical and biochemical parameters took place more often in the patient groups that had a care plan and even more with increasing number of care plan entries. The gap between guidelines and clinical practice in monitoring has previously been reported. In a recent Norwegian study, monitoring of LDL, sBP and HbA1c levels were observed within a year in 84.4%, 87.4% and 86.4% of patients with T2D in primary care, respectively, but only 30.3% of patients were tested for albuminuria [2]. Therefore, our finding that care plans are related to improved monitoring is important to clinical practice. Previously, a patient-empowerment programme was found to reduce the general outpatient clinic utilisation rate in patients with T2D in Hong Kong [19], but we do not know of any previous study to evaluate the association of care planning integrated into normal care to monitoring frequency in real-life in northern Europe.

In general, personalised care planning has previously found to be associated with some improvements in clinical and biochemical outcomes in patients with T2D [6]. More specifically, care planning according to the Finnish care plan form has previously been found to be associated with better clinical and biochemical outcomes [9]. We observed significant (but clinically relatively minor) differences between the groups in reaching the target goal of sBP. However, the LDL level improved only in the group with ≥ 3 care plan entries and the HbA1c level improved only in the group with 1–2 care plan entries. More care plan entries reflected longer follow-up periods and thus, longer diabetes durations, which worsened diabetic control [20]. Another study from Finland showed that the HbA1c levels fluctuated from year to year while the LDL levels decreased steadily [21]. An increasing number of consultations also implies the possibility that the doctor prescribed statins that improve LDL levels. However, T2D requires improvements in all its clinical outcomes. In the same study from Finland, the proportions of patients whose HbA1c and LDL were measured yearly were 75–78% and 67–69%, respectively, with relatively minor overall changes over time [21]. According to our results, monitoring was more scarcely performed in the usual care group than in either of the care plan groups, which may indicate that the actual clinical outcomes in the usual care group may be even worse.

In our study, the prescription of cardiovascular and antihyperglycemic medication was more frequent in care plan groups compared to usual care. Personalised care planning has previously been found to be associated with the more frequent use of metformin [11] and statins [9] compared to usual care. Our results add to the knowledge of the association between personalised care plans and cardiovascular-preventive and other hyperglycaemic medication. Our data also show that better achievement of treatment goals when using the Finnish care plan form as a tool in treatment are found. According to our results, personalised care planning might be associated with more intensive prescription of diabetes-related medication. The results are consistent with other studies on clinical results when patients have a care plan [22].

Our finding of the difference between care plan groups in the utilisation of different types of primary care physician services is of interest. Patients with a care plan utilised more planned diabetes consultations and had significantly fewer unplanned other contacts compared to the patients in the ‘no care plan’ group. This finding could partly be explained by the difference between the groups in monitoring frequency of the clinical parameters and prescription of preventive medication, which furthered the control of clinical and biochemical outcomes. This is in line with other studies where non-attendance at planned diabetes consultations resulted in poorer clinical outcomes [23]. In contrast, during unplanned contacts caregivers concentrated more on current incidental health problems instead of a holistic approach to a patient's long-term treatment. The planned health care utilisation supports continuity of care, which would be beneficial for both patients and caregivers.

In our study, the patient characteristics differed by care plan groups at baseline. Patients who did not receive a care plan were significantly older, had lower BMI and BP and had worse kidney function compared to patients with care plans. They were also prescribed preventive medications less commonly than the care plan groups. We do not know of any previous study that has described the characteristics of patients with T2D and without a written personalised care plan in clinical practice. The patient groups that benefit most from the care planning need to be recognised. It remains unclear if the clinicians are undertaking care planning for the patients who benefit most from it. We believe that help from clinical decision support systems would bring care planning to the front line in general practice and lessen the number of patients with T2D without a care plan [24,25].

### Meaning of the study

The Finnish structured care plan form is an easy to access, practical, low-intensity tool for care providers. Compared to many other care planning interventions, it can be performed by a single clinician or a nurse to be integrated into routine care in a variety of healthcare systems. We hypothesise that the structured care plan form guides caregivers towards practicing more patient-centric- yet evidenced-based-medicine- when treating patients with T2D, and it improves shared decision making in practice. It may also diminish the current problems caused by decreased continuity of care in primary care [26,27]. Nevertheless, the implementation of this method involves significant organisational cultural change. The results of our study confirm the relevance of personalised care planning in a real-world setting, and therefore helps caregivers and organisations to overcome the barriers to its implementation. Future studies should address the characteristics of the patients with T2D who would benefit most from a care plan. Furthermore, more research on both the barriers and enablers of the implementation of care plans in primary care is warranted.

## Supplementary Material

Supplemental Material
