# Supplementary material for: Association of personalised care plans with monitoring and control of clinical outcomes, prescription of medication and utilisation of primary care services in patients with type 2 diabetes: an observational real-world study
Source: Scand J Prim Health Care. 2022 Feb 11;40(1):39–47. doi: 10.1080/02813432.2022.2036458 (PMC9090399; doi:10.1080/02813432.2022.2036458)
Supplement: Supplemental Material [file IPRI_A_2036458_SM4037.docx]

**Supplement: A structured individualised care plan established according to Finnish national instructions, obligatory headlines being bolded.**

**Title: The health and care plan**

**Personal data of the patient**

**The compiler of the care plan**

**Date of documentation**

**Requirement/need for health care**

Narrative description of patient’s (or both patient’s and caregiver’s) perception of his/her current health problems for which the care plan has been established. The issues which patient feels to be insignificant are not addressed.

Goals of health care

Realistic targets, on which patient is able to commit, discussed and agreed upon by the patient and the caregiver, described narratively. Takes into account local processes and recommendations. Goals can be set by date after which they can be re-evaluated.

*For example:*

Maintenance of blood pressure: under 135/80 mmHg in home measurements (currently 142/79 mmHg)

Implementation and means

Detailed description, including i.e. recommended self management, or services provided by health care          system.

Support, follow-up and evaluation

I.e. settled date and type of next follow-up contact

Additional information

I.e. diagnosis, prescribed medication
